# Necroptosis in Esophageal Squamous Cell Carcinoma: An Independent Prognostic Factor and Its Correlation with Tumor-Infiltrating Lymphocytes

**DOI:** 10.3390/cancers13174473

**Published:** 2021-09-05

**Authors:** Takuro Yamauchi, Fumiyoshi Fujishima, Masatoshi Hashimoto, Junichi Tsunokake, Ryujiro Akaishi, Yusuke Gokon, Shunsuke Ueki, Yohei Ozawa, Toshiaki Fukutomi, Hiroshi Okamoto, Chiaki Sato, Yusuke Taniyama, Tomohiro Nakamura, Naoki Nakaya, Takashi Kamei, Hironobu Sasano

**Affiliations:** 1 Department of Surgery, Tohoku University Graduate School of Medicine, 1-1 Seiryo-machi, Aoba-ku, Sendai 980-8574, Japan; junichi-t.0-111@surg.med.tohoku.ac.jp (J.T.); ryujiro.a@surg.med.tohoku.ac.jp (R.A.); yusukegokon@surg.med.tohoku.ac.jp (Y.G.); s.ueki@surg.med.tohoku.ac.jp (S.U.); yohei.ozawa@surg.med.tohoku.ac.jp (Y.O.); t-fukutomi@med.tohoku.ac.jp (T.F.); hi-ok@surg.med.tohoku.ac.jp (H.O.); schiaki@surg.med.tohoku.ac.jp (C.S.); yusuketaniyama@med.tohoku.ac.jp (Y.T.); tkamei@surg.med.tohoku.ac.jp (T.K.); 2 Department of Pathology, Tohoku University Hospital, 1-1 Seiryo-machi, Aoba-ku, Sendai 980-8574, Japan; hashimoto@ped-surg.med.tohoku.ac.jp (M.H.); hsasano@patholo2.med.tohoku.ac.jp (H.S.); 3 Department of Pediatric Surgery, Tohoku University Graduate School of Medicine, 1-1 Seiryo-machi, Aoba-ku, Sendai 980-8574, Japan; 4 Department of Health Record Informatics, Tohoku Medical Megabank Organization, Tohoku University, 2-1 Seiryo-machi, Aoba-ku, Sendai 980-8573, Japan; tnakamura@megabank.tohoku.ac.jp; 5 Department of Preventive Medicine and Epidemiology, Tohoku Medical Megabank Organization, Tohoku University, 2-1 Seiryo-machi, Aoba-ku, Sendai 980-8573, Japan; naoki.nakaya.c2@tohoku.ac.jp

**Keywords:** esophageal squamous cell carcinoma, neoadjuvant chemotherapy, necroptosis, mixed lineage kinase domain-like protein, phosphorylated mixed lineage kinase domain-like protein

## Abstract

**Simple Summary:**

Necroptosis is a regulated form of necrotic cell death that plays pivotal roles in cancer biology, including tumorigenesis, metastasis, and cancer immunity. However, the significance of necroptosis in esophageal squamous cell carcinoma has remained largely unknown. In addition, its correlation with the tissue microenvironment has not yet been explored. In this study, we first investigated the diagnostic and prognostic significance of mixed lineage kinase domain-like protein (MLKL) and phosphorylated MLKL (pMLKL), both of which are currently considered the most reliable markers for detecting necroptosis. We also investigated the correlations between the status of MLKL/pMLKL and tumor-infiltrating lymphocytes) in esophageal squamous cell carcinoma patients.

**Abstract:**

Necroptosis is a pivotal process in cancer biology; however, the clinical significance of necroptosis in esophageal squamous cell carcinoma (ESCC) has remained unknown. Therefore, in this study, we aimed to verify the potential involvement of necroptosis in the clinical outcome, chemotherapeutic resistance, and tumor microenvironment of ESCC. Mixed lineage kinase domain-like protein (MLKL) and phosphorylated MLKL (pMLKL) were immunohistochemically examined in 88 surgically resected specimens following neoadjuvant chemotherapy (NAC) and 53 pre-therapeutic biopsy specimens, respectively. Tumor-infiltrating lymphocytes (TILs) were also evaluated by immunolocalizing CD3, CD8, and forkhead box protein 3 (FOXP3) in the residual tumors after NAC. High pMLKL status in the post-NAC resected specimens was significantly correlated with worse prognosis in ESCC patients. Multivariate analysis demonstrated that a high pMLKL status was an independent prognostic factor. In pre-NAC biopsy specimens, a high pMLKL status was significantly associated with a lower therapeutic efficacy. CD8+ TILs were significantly lower in the high-pMLKL group. FOXP3+ TILs were significantly higher in both high-MLKL and high-pMLKL groups. We first demonstrated pMLKL status as an independent prognostic factor in ESCC patients. Our study revealed the possible involvement of necroptosis in the immunosuppressive microenvironment, resulting in the attenuated therapeutic efficacy of NAC and eventual adverse clinical outcomes in ESCC.

## 1. Introduction

Esophageal cancer is the eighth most common cancer and the sixth leading cause of cancer-related death worldwide [1]. Esophageal squamous cell carcinoma (ESCC) is the most common esophageal cancer in Japan, and the standard treatment for patients with locally advanced ESCC is radical surgical resection following neoadjuvant chemotherapy (NAC) [2]. However, it is also true that the therapeutic efficacy of NAC exhibits large individual differences, and non–responders experience adverse effects before surgery without any clinical benefit [3,4]. Therefore, it is important to accurately predict the therapeutic response before the start of chemotherapy in order to establish the optimal treatment strategy for individual ESCC patients. 

Necroptosis, a molecularly regulated cell death, demonstrates all the morphological features of necrosis, including cell swelling, collapse of plasma membrane, and release of intracellular contents, all of which could subsequently induce secondary inflammatory responses [5,6,7]. Several studies on cell lines, animal models, and human tissue have indicated that necroptosis is a pivotal process in cancer biology, including tumorigenesis, metastasis, and cancer immunity [8,9].

The necroptosis pathway is initiated by the activation of receptor interacting protein kinase 1 (RIP1), which then interacts with RIP3, resulting in the formation of a necrosome complex [10,11,12]. The necrosome subsequently induces phosphorylation of mixed lineage kinase domain-like protein (MLKL) to produce phosphorylated MLKL (pMLKL), which oligomerizes and translocates into the plasma membrane, where it mediates the disruption of plasma membrane permeability by activating ion channels or forming pore structures, finally resulting in the induction of necrotic cell death [13,14,15]. In addition, pMLKL is currently the most reliable marker for detecting necroptosis [15]. The induction of necroptosis can also induce secondary inflammatory responses by releasing intracellular damage-associated molecular patterns (DAMPs) [16]. This inflammation eventually activates some signaling pathways, including NF-κB or MAPK pathways; these are all involved in tumor progression [17]. Liu et al. demonstrated that necroptotic pathways played a pivotal role in the activity of NF-κB, resulting in tumor progression; they also reported that high levels of pMLKL were associated with poor prognosis in patients with esophageal or colon cancers [18]. Seifert et al. also demonstrated that necroptosis could promote tumorigenesis by releasing chemokine (C-X-C motif) ligand 1 (CXCL1) and 130 kDa Sin3-associated polypeptide (SAP130) to induce an immunosuppressive tumor microenvironment (TME) in pancreatic ductal adenocarcinoma [19].

The induction of necroptosis in carcinoma cells modulates the TME and regulates anti-tumor immunity [20]. Tumor-infiltrating lymphocytes (TILs) play the main role in anti-tumor immunity, and its status has been identified as a prognostic marker in several human malignancies [21,22,23]. In addition, TILs were reported to be correlated with the therapeutic efficacy of chemotherapy in ESCC, triple-negative breast cancer (TNBC), and urothelial carcinoma of the urinary bladder [24,25,26]. Therefore, necroptosis in cancer tissue is reasonably postulated to lead to therapeutic resistance; however, the correlation between the extent of necroptosis and the efficacy of chemotherapy remains virtually undetermined. It has, thus, become important to verify the potential biomarkers that could predict the therapeutic efficacy of chemotherapy and the clinical outcomes of ESCC patients. Therefore, in this study, we aimed to investigate the following in ESCC patients: (1) the status of MLKL and pMLKL in pre- and post-NAC specimens, and their correlation with the clinical outcome/therapeutic efficacy of NAC; and (2) the correlation between these necroptotic markers and TILs, examined by CD3, CD8, and FOXP3 immunolocalization in ESCC patients.

## 2. Materials and Methods

### 2.1. Patients and Tissue Specimens

In this study, 88 ESCC patients were examined. All patients underwent surgical resection with regional lymph node dissection following NAC based on the Japanese Clinical Oncology Group 9907 (JCOG9907) protocol at Tohoku University Hospital (Sendai, Japan) from 2009 to 2016 [3]. Of the 88 cases, pre-NAC biopsy specimens were obtained for evaluation in 53 cases. Tumors were pathologically classified according to the Union for International Cancer Control TNM staging system (8th edition) for esophageal carcinoma [27]. The histological classification for the effects of NAC was determined as follows: Grade 0, ineffective (no cytological or histological therapeutic effects detected in the primary lesion); Grade 1, slightly effective (Grade 1a, necrosis, fibrosis or granulomatous changes detected in less than one-third of the residual tumor lesion; Grade 1b, in one-third to two-thirds of the lesion); Grade 2, moderately effective (necrosis, fibrosis or granulomatous changes detected in more than two-thirds of the lesion, although viable residual tumor cells were histologically detected); and Grade 3, markedly effective (no viable residual tumor cells) [28]. However, none of the 88 patients with ESCC demonstrated histological Grade 3 or no residual tumors in the post-NAC resected specimens examined. We also evaluated the therapeutic efficacy of NAC according to the Response Evaluation Criteria in Solid Tumors (RECIST) version 1.1 [29]. The patients were classified under the following four categories: complete response (CR), partial response (PR), stable disease (SD), and progressive disease (PD). In total, 12 patients were excluded from further evaluation based on RECIST due to difficulties in measuring the diameter of the primary lesions.

In addition, we also retrieved the data of pathological CR (pCR) cases, defined as histological grade 3 in post-NAC resected specimens, and obtained pre-therapeutic biopsy specimens from 8 patients, from 2009 to 2021. All eight patients underwent equivalent radical resection following NAC.

The starting point of survival in ESCC patients examined in this study was the date of surgery. The endpoints for overall survival (OS) and disease-free survival (DFS) rates were patient death, recurrence, or the last censor date. The median length of clinical follow-up for censored patients was 5.0 (range, 2.2–5.0) years.

### 2.2. Neoadjuvant Chemotherapy and Surgery

Preoperative chemotherapy, performed according to the JCOG 9907 protocol [3], was administered in conjunction with intravenous infusion of cisplatin (80 mg/m^2^) on days 1 and 22, and continuous intravenous infusion of 5-fluorouracil (800 mg/m^2^/day) over 24 h on days 1–5 and 22–26. Subsequently, thoracoscopic esophagectomy, gastric tube reconstruction, and cervical esophagogastric anastomosis were performed with regional lymph node dissection.

### 2.3. Immunohistochemical Staining

Tissue specimens were fixed in 10% neutral formalin and embedded in paraffin. Representative paraffin blocks from each specimen were selected for immunohistochemistry and all contained invasive edges and viable tumor cells after careful histopathological evaluation in hematoxylin and eosin-stained tissue slides. Information regarding immunohistochemical procedures and primary antibodies is summarized in Table S1.

Tissue sections (4 μm thick) were serially cut and placed on clean glass slides. The sections were deparaffinized with xylene and rehydrated using graded ethanol solutions. Heat-induced antigen retrieval was performed as described in Table S1. After blocking endogenous peroxidases, the sections were treated with primary antibodies overnight at 4 °C. Subsequently, the sections were incubated with HRP-polymer secondary antibodies (EnVision FLEX Kit FLEX/HRP, Agilent Technologies, Santa Clara, CA, USA) at 24 °C for 30 min. Only FOXP3 staining was performed with biotinylated anti-mouse immunoglobulin (Histofine Kit; Nichirei Bioscience, Tokyo, Japan) as a secondary antibody and peroxidase-labeled streptavidin (Histofine Kit; Nichirei Bioscience) at 24 °C for 30 min. The antigen–antibody reactions were visualized using 3.3-diaminobenzidine and counterstaining was performed using hematoxylin and eosin.

Each stained tissue section was independently evaluated by two observers (TY and FF) who had no clinicopathological information of the patients. MLKL immunoreactivity was semi-quantitatively assessed using a histological score (H-score), which was calculated by multiplying the percentage of cytoplasm-stained tumor cells and the staining intensity score (0, negative; 1, weak; 2, moderate; and 3, marked), ranging from 0 to 300 [30]. pMLKL immunoreactivity was assessed using a labeling index by counting the proportion of cytoplasm- or plasma membrane-stained tumor cells [30]. We selected five non-overlapping fields under a light microscope at 400× magnification, with each field containing more than 100 viable tumor cells. The cutoff values of MLKL immunoreactivity were tentatively determined as their median H-score, and independently established thresholds for pre-NAC biopsy and post-NAC resected specimens as follows: “40” for post-NAC MLKL and “70” for pre-NAC MLKL. We also determined the optimal cutoff values of pMLKL for the survival outcome of the patients by drawing the receiver operating characteristic curve, and independently established thresholds for pre-NAC biopsy and post-NAC surgically resected specimens as follows: “3.9%” for post-NAC pMLKL and “2.1%” for pre-NAC. Specimens were tentatively classified into low or high expression groups based on the thresholds of each marker examined.

In this study, we evaluated TILs according to the recommendations of the International TIL Working Group [31]. TILs were assessed within the borders of the invasive tumor. First, the tumor borderlines were carefully confirmed by examination of hematoxylin and eosin-stained tissue slides under light microscopy at 100× magnification. Each section was then examined at 400× magnification, and five non-overlapping fields with abundant TILs were selected from the stromal components of tumor tissues that were near the viable tumor cells (Figure S1). Insufficient stromal components were detected in six cases that were subsequently excluded from the TIL assessment. The numbers of CD3+, CD8+, and FOXP3+ T lymphocytes were counted per field, and the mean number of TILs was the final result of the evaluation.

### 2.4. Statistical Analysis

All statistical analyses were performed using the EZR version 1.38 (Saitama Medical Center, Jichi Medical University; Saitama, Japan) [32]. Continuous data were analyzed using the Mann–Whitney U test. The relationship and correlation between two variables were analyzed using Fisher’s exact test. OS and DFS curves were calculated using the Kaplan–Meier method, and the log-rank test was performed for comparisons. Univariate and multivariate analyses were performed using the Cox proportional hazards model. Statistical significance was set at *p* < 0.05.

## 3. Results

### 3.1. Post-NAC MLKL and pMLKL Status and Their Correlation with Clinicopathological Factors in ESCC Patients

**Figure 1:**
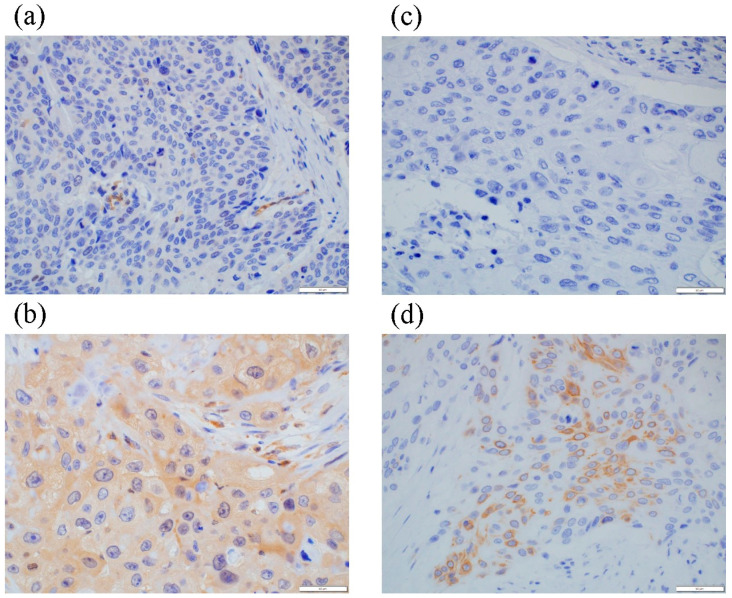
Representative illustrations of MLKL and pMLKL immunohistochemistry. (**a**) Low MLKL and (**b**) high MLKL status showing representative cases of diffuse and marked immunoreactivity in the cytoplasm of carcinoma cells. (**c**) Low pMLKL and (**d**) high pMLKL status showing representative cases demonstrating pMLKL immunoreactivity in the cytoplasm and plasma membrane of carcinoma cells.

Representative microscopic images of MLKL and pMLKL immunohistochemistry are demonstrated in Figure 1. We examined the correlation of these immunoreactivities with clinicopathological factors of the patients, including age and sex, pT, pN, and pStage; tumor differentiation; resected margin; RECIST grade; and histopathological tumor regression grade (Table 1). In the surgically resected specimens following NAC, a high status of pMLKL (detected in 21.6% (19/88)) was significantly correlated with age (*p* = 0.029) and pT status (*p* = 0.009). However, no significant correlations were detected between pMLKL status and sensitivity to NAC (RECIST grade, *p* = 0.777; and histopathological tumor regression grade, *p* = 0.189). No significant correlations were detected between MLKL status and any clinicopathological factor.

### 3.2. Post-NAC MLKL and pMLKL Status and Their Correlation with the Clinical Outcome of ESCC Patients

**Figure 2:**
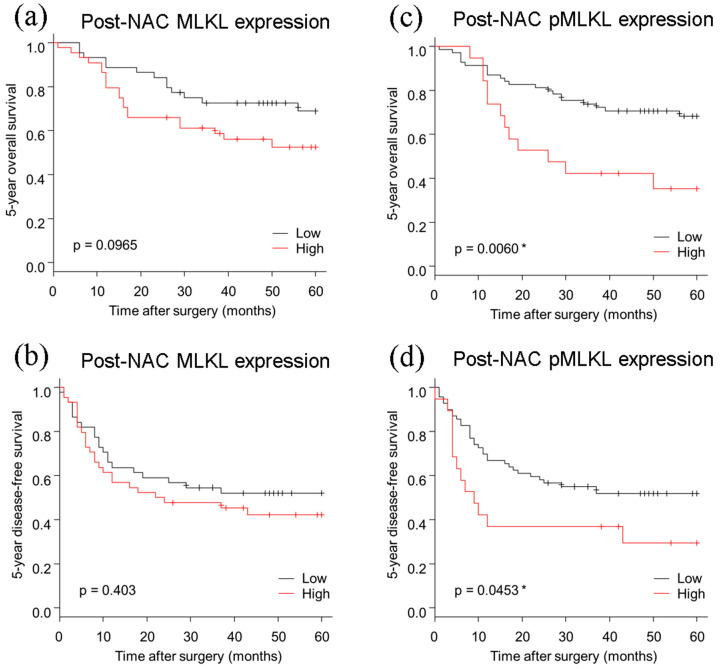
Kaplan–Meier curves based on post-NAC MLKL and post-NAC pMLKL status. (**a**,**b**) no significant difference was detected in post-NAC MLKL status for the 5 year OS and the 5 year DFS. (**c**) The 5 year OS was significantly worse in patients with high pMLKL than in those with low pMLKL status. (**d**) The 5 year DFS was significantly worse in those with high pMLKL status than in those with low pMLKL status. OS, overall survival; DFS, disease-free survival; NAC, neoadjuvant chemotherapy. * Statistical significance.

The 5 year OS (*p* = 0.0060) and DFS (*p* = 0.0453) of ESCC were both significantly lower in the high pMLKL group (Figure 2). In contrast, no significant correlations were detected between the eventual clinical outcomes and MLKL status (OS, *p* = 0.0965; DFS, *p* = 0.4030). Univariate analysis revealed that the OS rate was significantly associated with pT (*p* = 0.037), pN (*p* = 0.010), pStage (*p* = 0.004), resection margin (*p* < 0.001), histopathological tumor regression grade (*p* = 0.017), and high pMLKL status (*p* = 0.009) (Table 2). Multivariate analysis revealed that pN (*p* = 0.018), resection margin (*p* = 0.013), and high pMLKL expression (*p* = 0.027) were independent prognostic factors (Table 3).

### 3.3. Pre-NAC MLKL and pMLKL and Their Correlation with Clinicopathological Factors of ESCC Patients

In the biopsy specimens, a high pMLKL status was detected in 26.4% (14/53) of patients. There was a high concordance of pMLKL between pre- and post-NAC tumor tissue specimens (81.1%). However, there was a 35.8% discordance in MLKL status. The high status of pMLKL in pre-NAC tumor specimens was significantly correlated with the histopathological tumor regression grade (*p* = 0.013) (Table 4). Pre-NAC MLKL status was also significantly correlated with pN (*p* = 0.042) and tumor differentiation (*p* = 0.010).

We also explored the status of MLKL and pMLKL in pre-NAC endoscopic biopsy ESCC specimens in eight patients who developed pCR. A high pMLKL status was not detected in any of the eight patients. When compared to the non-pCR group (*N* = 53), pMLKL status tended to be lower in the pCR group (*N* = 8), although the difference was not statistically significant (Figure S2). In addition, there were no significant differences in MLKL status between the two groups.

### 3.4. Pre-NAC MLKL and pMLKL Status and Their Correlation with Clinical Outcome of ESCC Patients

**Figure 3:**
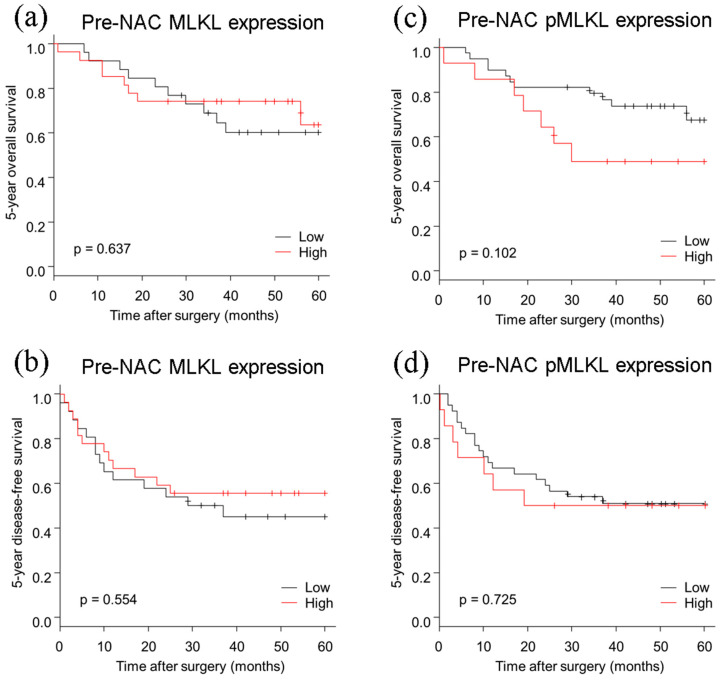
Kaplan–Meier curves based on pre-NAC MLKL and pre-NAC pMLKL status. (**a**,**b**) No significant difference was detected in pre-NAC MLKL status for the 5 year OS and DFS. (**c**) The 5 year OS tended to be worse in patients with high pMLKL status than in those with low pMLKL, but this difference did not reach statistical significance. (**d**) No significance was detected in pre-NAC pMLKL status for the 5 year DFS. Abbreviations: MLKL, mixed lineage kinase domain-like protein; pMLKL, phosphorylated MLKL; OS, overall survival; DFS, disease-free survival; NAC, neoadjuvant chemotherapy.

The 5-year OS rate of ESCC patients with high pre-NAC pMLKL levels tended to be shorter than that of patients with low levels, although the difference was not statistically significant (*p* = 0.102) (Figure 3). In addition, there were no significant correlations between the eventual clinical outcomes of ESCC patients and MLKL status (OS: *p* = 0.637, DFS: *p* = 0.554). Univariate analysis revealed that the correlation between the 5 year OS and pre-NAC pMLKL status was detected, although it was not statistically significant (*p* = 0.111) (Table 5). 

### 3.5. Correlations between the Necroptotic Biomarkers and TILs in ESCC

**Figure 4:**
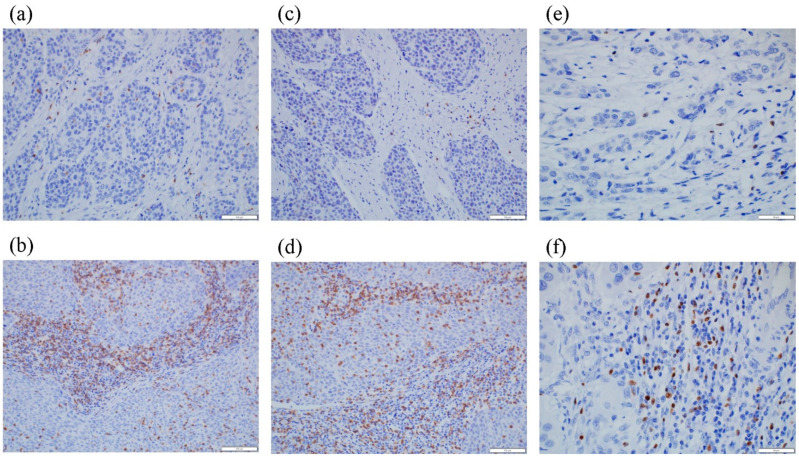
Representative illustrations of tumor-infiltrating lymphocytes (TILs). (**a**) Low CD3+ TILs, and (**b**) high CD3+ TILs. (**c**) Low CD8+ TILs, and (**d**) high CD8+ TILs. (**e**) Low FOXP3+ TILs, and (**f**) high FOXP3+ TILs.

**Figure 5:**
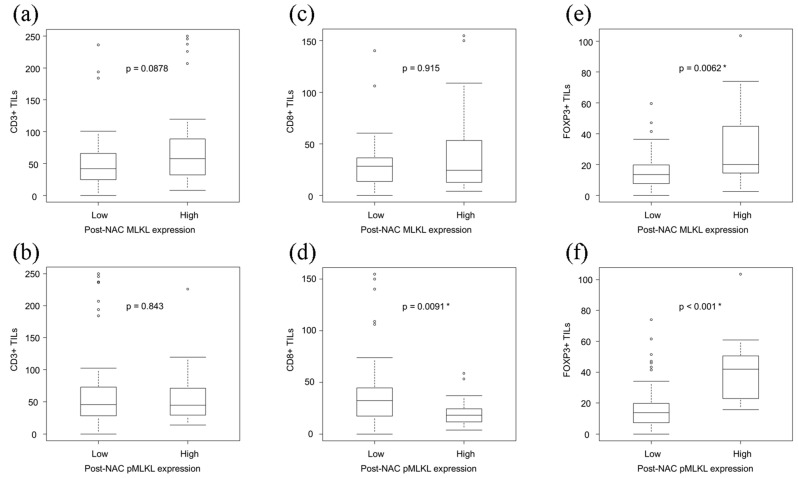
Representative illustrations of tumor-infiltrating lymphocytes (TILs). (**a**) Low CD3+ TILs, and (**b**) high CD3+ TILs. (**c**) Low CD8+ TILs, and (**d**) high CD8+ TILs. (**e**) Low FOXP3+ TILs, and (**f**) high FOXP3+ TILs. * Statistical significance.

We then examined the correlations between the status of the necroptotic markers and the density of TILs in post-NAC residual tumors. Representative images of CD3, CD8, and FOXP3 staining are illustrated in Figure 4. Patients with high pMLKL status harbored significantly lower CD8+ TILs than those with low (*p* = 0.0091) (Figure 5). In addition, those with high pMLKL levels harbored significantly higher levels of FOXP3+ TILs (*p* < 0.001). High MLKL status was also significantly correlated with higher levels of FOXP3+ TILs (*p* = 0.0062). However, there were no significant correlations between MLKL/pMLKL status and CD3+ TILs.

## 4. Discussion

This is the first study to show the clinical significance of necroptosis in ESCC. The findings demonstrated that the status of pMLKL in resected tissue specimens was significantly associated with pT status and poor prognosis in ESCC patients undergoing NAC. In addition, the extent of necroptosis in carcinoma cells was closely related to the invasive phenotypes and poor prognosis in ESCC patients. The prognostic significance of necroptosis in tumor tissue remains controversial; however, necroptosis of tumor cells promotes tumor development and acts as a poor prognostic factor in several human malignancies. According to Jiao et al., necroptosis was detected around necrotic foci in tissues of both mouse MMVT-PyMT and human breast cancer and demonstrated a critical role in tumor progression and metastasis [33]. Li et al. also reported that necroptosis could promote migration and invasion of head and neck squamous cell carcinoma (HNSCC) cells, and that the extent of necroptosis was a specific prognostic factor in HNSCC [34]. Our present study provided additional evidence for the clinical significance of necroptosis in ESCC patients undergoing NAC.

In addition, we examined the status of MLKL and pMLKL in pre- and post-NAC specimens to explore their correlation with the therapeutic efficacy of NAC. The high status of pMLKL in pre-NAC biopsy specimens was significantly associated with a lower histopathological tumor regression grade. Furthermore, lower histopathological tumor regression grade, but not RECIST grade, was significantly associated with poor prognosis of ESCC patients examined. Our findings also suggested that the extent of necroptosis could attenuate the therapeutic efficacy of chemotherapy, resulting in tumor progression and subsequent adverse clinical outcomes in patients. In addition, the therapeutic efficacy of NAC could be predicted by studying the status of pMLKL in pre-NAC biopsy specimens and all the eight patients diagnosed with pCR demonstrated low pMLKL in pre-NAC biopsy specimens. No significant associations were detected between high pMLKL status in pre-NAC biopsy specimens and lower RECIST grade could be due to the relatively small number of biopsy specimens examined in our study, and that the indeterminate cases were biased toward the low pMLKL group. Therefore, further investigations are required to clarify the correlation between pMLKL status and the therapeutic efficacy of NAC.

Despite the possible significance of necroptosis in the therapeutic efficacy of NAC in pre-NAC biopsy specimens, there were no significant associations in post-NAC resected specimens. This may be due to biological alterations in tumor tissue caused by chemotherapy. Several chemotherapeutic drugs induced necroptosis under certain conditions. For example, cisplatin induced necroptosis in apoptosis-resistant ESCC cells [35]. 5-fluorouracil induced necroptosis in human colon carcinoma cells when caspase activity was inhibited [36]. Li et al. reported that the expression levels of pMLKL were influenced by NAC in pancreatic ductal adenocarcinoma [37]. Acute massive necroptosis induced by chemotherapy could, therefore, promote inflammatory responses and provide immunogenic effects on anti-tumor immunity [38]. In contrast, mild degrees of chronic necroptosis, which could occur when tumor cells were exposed to metabolic stress, was reported to suppress anti-tumor immunity by releasing immunosuppressive molecules, resulting in the modulation of the tissue microenvironment and tumor development [39]. Necroptosis of tumor cells therefore played dual conflicting roles, namely immunogenic or immunosuppressive effects on anti-tumor immunity [39,40]. In our present study, whether the expression of pMLKL detected in resected specimens was induced after or before chemotherapy has remained unknown; therefore, the definitive roles of necroptosis in ESCC could be influenced by chemotherapy itself.

We examined the correlations between the biomarkers of necroptosis and the status of TILs in the resected tissue specimens and confirmed their significant associations with the immunological profile of ESCC. Our findings also suggested that necroptosis could play a pivotal role in anti-cancer immunity in ESCC patients. For instance, regulatory T cells (T-regs or FOXP3+ T cells) are immunosuppressive subsets of CD4+ T cells, and hinder the protective immune responses in cancer-bearing hosts [41]. In contrast, cytotoxic T cells (CTL or CD8+ T cells) are generally recognized as a protective immune subset that targets cancer cells [23]. CD8+ TILs are also key prognostic markers in many human malignancies [22,23,24]. In addition, tumor-infiltrating CTLs have also been reported as a predictor of pCR after neoadjuvant chemoradiotherapy (NACRT) in ESCC patients [42]. In contrast, tumor-infiltrating T-regs in residual tumors are a negative predictor of NAC or NACRT efficacy in ESCC patients [25,43]. In pancreatic ductal carcinoma (PDA), the necroptotic pathway was reported to induce immunosuppressive effects in the TME. Seifert et al. reported that PDA cells could release CXCL1 through the necroptotic pathway, which subsequently induced the infiltration of immunosuppressive myeloid-derived suppressor cells (MDSCs) and M2-like macrophages [19]. Furthermore, the nuclear factor SAP130, released from PDA cells, could bind to macrophage-inducible Ca2+-dependent lectin receptor (Mincle). Subsequently, this Mincle ligation could reduce the cytotoxic T lymphocyte infiltration and activation [19]. In addition, a high level of potassium released from necrotic tumor cells inhibits both CD4+ and/or CD8+ T lymphocyte activation [44]. Consistent with the above, our findings indicated that necroptotic cancer cells could indeed induce immunosuppressive TME, hinder anti-tumor immunity, and promote tumor progression; however, further investigations are required for clarity.

Our study has certain limitations in the evaluation of the immunohistochemical analysis of the pre-NAC biopsy samples. Considering intratumoral heterogeneity, the biopsy samples represented carcinoma tissues located predominantly on the tumor surface and therefore did not necessarily reflect the features of the whole tumor, including the invasive areas. In addition, intratumoral heterogeneity of the markers examined in this study could have confounded the results.

## 5. Conclusions

In conclusion, results of our present study demonstrated that the status of pMLKL in ESCC patients undergoing NAC was significantly associated with the clinical outcome. In addition, we also demonstrated that necroptosis could be involved in immunosuppressive TME and the therapeutic effects of chemotherapy in patients with ESCC. Therefore, the potential utility of pMLKL as a predictable marker could help avoid unnecessary NAC in non–responders.

## Figures and Tables

**Table 1:** Post-NAC expression status of the markers and its correlation with clinicopathological factors.

Variable	*N*	Post-NAC MLKL		*p*	Post-NAC pMLKL		*p*
		High	Low		High	Low	
	88	44	44		19	69	
Age (years)							
≥65	57	25	32	0.180	8	49	0.029 *
<65	31	19	12		11	20	
Gender							
Male	74	37	37	1.000	13	61	0.069
Female	14	7	7		6	8	
pT							
pT1-T2	37	17	20	0.666	3	34	0.009 *
pT3-T4	51	27	24		16	35	
pN							
pN0	24	10	14	0.473	3	21	0.255
pN1-N3	64	34	30		16	48	
Pathological stage							
Stage I/II	31	13	18	0.372	4	27	0.181
Stage III/IV	57	31	26		15	42	
Differentiation							
Well, moderate	76	37	39	0.200	18	58	0.661
Poor	10	7	3		1	9	
Unclassifiable	2	0	2		0	2	
Resection margin							
R0	80	39	41	0.713	18	62	1.000
R1	8	5	3		1	7	
RECIST grade							
CR/PR	22	14	8	0.206	6	16	0.777
SD/PD	54	24	30		13	41	
Indeterminate	12	6	6		0	12	
Histological NAC efficacy							
Ineffective (Grade 0-1a)	51	28	23	0.388	14	37	0.189
Effective (Grade 1b-2)	37	16	21		5	32	

Abbreviation: NAC—neoadjuvant chemotherapy; MLKL—mixed lineage kinase domain-like protein; pMLKL—phosphorylated MLKL; RECIST, Response Evaluation Criteria in Solid Tumors; CR, complete response; PR, partial response; SD, stable disease; PD, progressive disease. * Statistical significance.

**Table 2:** Univariable analysis of patients’ 5 year overall survival.

Variable	Person-Year	Event	HR (95% CI)	*p*
Age (years)				
<65	101	11	ref	
≥65	194	22	1.08 (0.52–2.23)	0.830
Gender				
Female	42	7	ref	
Male	253	26	0.63 (0.27–1.45)	0.280
pT				
pT1-2	138	9	ref	
pT3-T4	157	24	2.26 (1.05–4.87)	0.037 *
pN				
pN0	98	3	ref	
pN1-N3	198	30	4.73 (1.44–15.53)	0.010 *
Pathological stage				
Stage I/II	129	5	ref	
Stage III/IV	167	28	4.08 (1.57–10.59)	0.0039*
Differentiation				
Poor	29	3	ref	
Well, moderate	258	30	1.24 (0.38–4.08)	0.720
Resection margin				
R0	282	26	ref	
R1	14	7	5.03 (2.15–11.8)	<0.001 *
RECIST grade				
CR/PR	77	5	ref	
SD/PD	175	24	2.13 (0.81–5.59)	0.120
Histological NAC efficacy				
Ineffective (Grade 0–1a)	158	25	ref	
Effective (Grade 1b–2)	138	8	0.38 (0.17–0.84)	0.017 *
post-NAC MLKL				
Low	162	13	ref	
High	134	20	1.79 (0.89–3.60)	0.100
post-NAC pMLKL				
Low	245	21	ref	
High	50	12	2.60 (1.27–5.31)	0.0087 *

Abbreviations: HR, hazard ratio; CI, confidence interval; NAC, neoadjuvant chemotherapy, MLKL, mixed lineage kinase domain-like protein; pMLKL, phosphorylated MLKL; RECIST, Response Evaluation Criteria in Solid Tumors; CR, complete response; PR, partial response; SD, stable disease; PD, progressive disease. * Statistical significance.

**Table 3:** Multivariable analysis of patients’ 5 year overall survival.

Variable	Person-Year	Event	HR (95% CI)	*p*
Age (years)				
<65	101	11	ref	
≥65	194	22	1.35 (0.63–2.92)	0.440
Gender				
Female	42	7	ref	
Male	253	26	0.64 (0.25–1.61)	0.344
pT				
pT1-T2	138	9	ref	
pT3-T4	157	24	1.30 (0.56–3.00)	0.546
pN				
pN0	98	3	ref	
pN1-N3	198	30	4.42 (1.29–15.20)	0.018 *
Resection margin				
R0	282	26	ref	
R1	14	7	3.43 (1.30–9.05)	0.013 *
Histological NAC efficacy				
Ineffective (Grade 0–1a)	158	25	ref	
Effective (Grade 1b–2)	138	8	0.52 (0.22–1.21)	0.127
post-NAC pMLKL				
Low	245	21	ref	
High	50	12	2.48 (1.11–5.57)	0.027 *

Abbreviations: HR, hazard ratio; CI, confidence interval; NAC, neoadjuvant chemotherapy; pMLKL, phosphorylated mixed lineage kinase domain-like protein. * Statistical significance.

**Table 4:** Pre-NAC expression status of the markers and its correlation with clinicopathological factors.

Variable	*N*	Pre-NAC MLKL		*p*	Pre-NAC pMLKL		*p*
		High	Low		High	Low	
	53	27	26		14	39	
Age (years)							
≥65	33	16	17	0.779	7	26	0.341
<65	20	11	9		7	13	
Gender							
Male	45	25	20	0.142	11	34	0.422
Female	8	2	6		3	5	
pT							
pT1-T2	28	13	15	0.586	6	22	0.534
pT3-T4	25	14	11		8	17	
pN							
pN0	17	5	12	0.042 *	2	15	0.180
pN1-N3	36	22	14		12	24	
Pathological stage							
Stage I/II	19	7	12	0.158	3	16	0.330
Stage III/IV	34	20	14		11	23	
Differentiation							
Well, moderate	45	20	25	0.010 *	12	33	1.000
Poor	7	7	0		2	5	
Unclassifiable	1	0	1		0	1	
Resection margin							
R0	50	25	25	1.000	14	36	0.557
R1	3	2	1		0	3	
RECIST grade							
CR/PR	16	8	8	0.540	3	13	0.195
SD/PD	28	17	11		11	17	
Indeterminate	9	2	7		0	9	
Histological NAC efficacy							
Ineffective (Grade 0–1a)	30	17	13	0.412	12	18	0.013 *
Effective (Grade 1b–2)	23	10	13		2	21	

Abbreviation: NAC, neoadjuvant chemotherapy, MLKL, mixed lineage kinase domain-like protein; pMLKL, phosphorylated MLKL; RECIST, Response Evaluation Criteria in Solid Tumors; CR, complete response; PR, partial response; SD, stable disease; PD, progressive disease. * Statistical significance.

**Table 5:** Univariate analysis of patients’ 5 year overall survival based on the pre-NAC status of the markers.

Variable	Person-Year	Event	HR (95% CI)	*p*
pre-NAC MLKL				
Low	86	10	ref	
High	88	8	0.80 (0.31–2.03)	0.638
pre-NAC pMLKL				
Low	137	11	ref	
High	38	7	2.17 (0.84–5.64)	0.111

Abbreviations: HR, hazard ratio; CI, confidence interval; NAC, neoadjuvant chemotherapy; MLKL, mixed lineage kinase domain-like protein; pMLKL, phosphorylated MLKL.

## Data Availability

The data used and/or analyzed in this study are available from the corresponding author. The data are not publicly available because of ethical restrictions.

## Supplementary Materials

The following are available online at https://www.mdpi.com/article/10.3390/cancers13174473/s1, Figure S1: Tumor-infiltrating lymphocytes (TILs) were assessed within the borders of the invasive tumor. Fields with abundant TILs were selected from the stromal components of tumor tissues (dashed line) and quantified at 400× magnification. Figure S2: Expression status of necroptotic biomarkers in the pCR (*N* = 8) and non-pCR groups (*N* = 53). (**a**) No significant differences were detected in the frequency of MLKL status between the two groups (*p* = 0.707). (**b**) The frequency of pMLKL tended to be lower in the pCR group in which a high pMLKL status was not detected, but this difference was not statistically significant (*p* = 0.180). Table S1: Characteristics of primary antibodies used in this study
