# Supplementary material for: Necroptosis in Esophageal Squamous Cell Carcinoma: An Independent Prognostic Factor and Its Correlation with Tumor-Infiltrating Lymphocytes
Source: Cancers (Basel). 2021 Sep 5;13(17):4473. doi: 10.3390/cancers13174473 (PMC8430921; doi:10.3390/cancers13174473)
Supplement: Supplementary file 1 [file cancers-13-04473-s001.zip › cancers-1359878-SI.pdf]

Supplementary Materials: Necroptosis in Esophageal Squamous Cell Carcinoma: An Independent Prognostic Factor and Its Correlation with Tumor-Infiltrating Lymphocytes

Takuro Yamauchi, Fumiyoshi Fujishima, Masatoshi Hashimoto, Junichi Tsunokake, Ryujiro Akaishi, Yusuke Gokon, Shunsuke Ueki, Yohei Ozawa, Toshiaki Fukutomi, Hiroshi Okamoto, Chiaki Sato, Yusuke Taniyama, Tomohiro Nakamura, Naoki Nakaya, Takashi Kamei and Hironobu Sasano

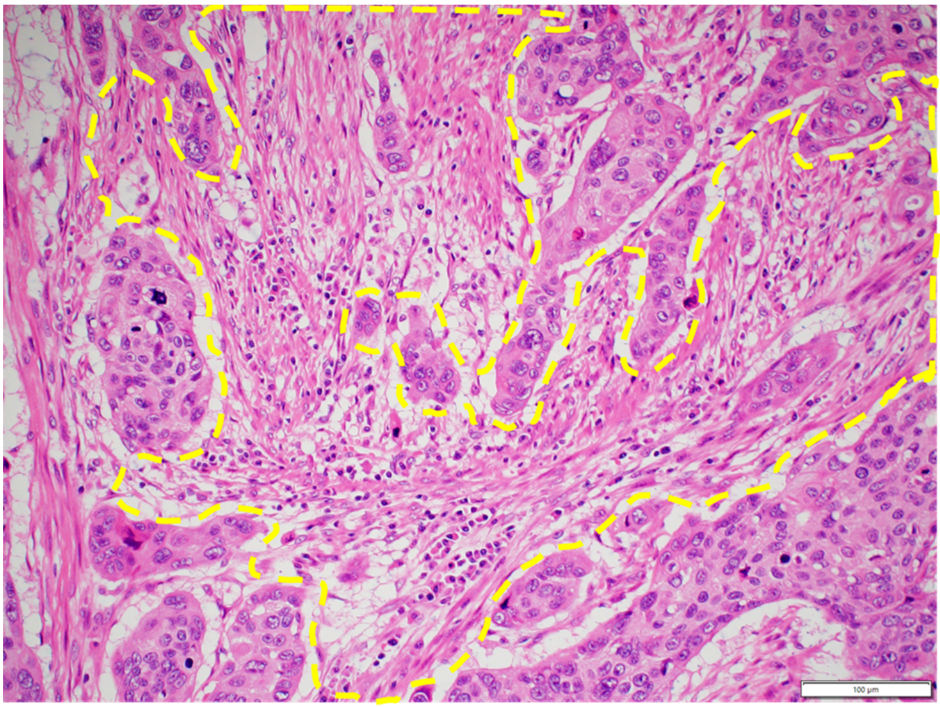

Figure S1. Tumor-infiltrating lymphocytes (TILs) were assessed within the borders of the invasive tumor. Fields with abundant TILs were selected from the stromal components of tumor tissues (dashed line) and quantified at 400× magnification.

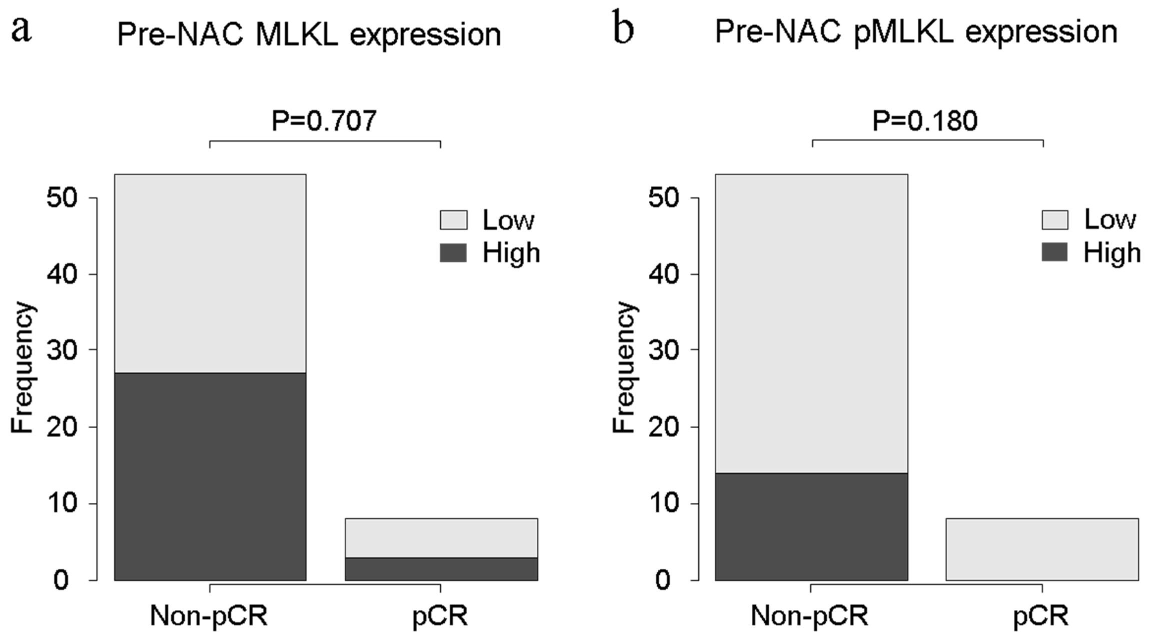

Figure S2. Expression status of necroptotic biomarkers in the pCR (N = 8) and non-pCR groups (N = 53). (**a**) No significant differences were detected in the frequency of MLKL status between the two groups (p = 0.707). (**b**) The frequency of pMLKL tended to be lower in the pCR group in which a high pMLKL status was not detected, but this difference was not statistically significant (p = 0.180).

Table S1. Characteristics of primary antibodies used in this study.

Antibody	Host	Clone	Vendor	Dilution	Antigen Retrieval	Buffer pH
MLKL	Rabbit	EPR17514	Abcam	1/2000	AC, 121℃, 5 min	9.0
pMLKL	Rabbit	EPR9514	Abcam	1/800	AC, 121℃, 5 min	9.0
CD3	Rabbit	Polyclonal	Dako	1/500	AC, 121℃, 5 min	9.0
CD8	Mouse	C8/144B	Dako	1/50	AC, 121℃, 5 min	7.0
FOXP3	Mouse	236A/E7	Abcam	1/200	AC, 121℃, 5 min	6.0
